# Modelling Spatial and Temporal Patterns of African Swine Fever in an Isolated Wild Boar Population to Support Decision-Making

**DOI:** 10.3389/fvets.2020.00154

**Published:** 2020-04-08

**Authors:** Simon Croft, Giovanna Massei, Graham C. Smith, David Fouracre, James N. Aegerter

**Affiliations:** National Wildlife Management Centre, Animal and Plant Health Agency, York, United Kingdom

**Keywords:** individual-based, real-world landscape, UK, wildlife management, contingency planning

## Abstract

African swine fever (ASF) is a highly contagious disease affecting all suids including wild boar. As the disease can damage commercial pig production and its circulation can threaten international trade, understanding the risks produced by free-living wild boar (as a wildlife reservoir) is important to ensure proportionate policies to exclude the disease, as well as an effective contingency response. The recent spread of the virus into Western Europe has produced concerns in many stakeholders including pig producers and national governments. Unlike in mainland Europe, where wild boar are widespread, in Britain, free-living populations have only recently re-established, and whilst these are still relatively small and isolated, they may provide a sufficient reservoir capable of sustaining disease and may thus present a continual source of infection risk to domestic pigs. This study focuses on one component of the risk produced by wild boar, specifically the distribution and persistence of virus in a landscape produced by the natural circulation of disease within wild boar. We used a spatial individual-based model run across a representation of a real landscape to explore the epidemiological consequences of an introduction of ASF into the Forest of Dean, currently hosting the largest population of wild boar in England. We explore various scenarios including variations in the prophylactic management of boar, as well as variations in reactive management (contingency response) following the detection of disease to evaluate their value in reducing this specific risk (presence of ASF virus of wild boar origin in the landscape). The abundance and distribution of wild boar is predicted to increase across our study extent over the next 20 years. Outbreaks of ASF are not predicted to be self-sustaining, with the median time to disease “burn-out” (no new infections) being 14 weeks. Carcass removal, as a tool in a package of reactive management, was of limited value in reducing the duration of outbreaks in this study. We suggest that useful predictions of some of the risks produced by ASF might be possible using only the distribution of the boar, rather than more difficult abundance or density measures.

## Introduction

African swine fever (ASF) is a highly contagious and virulent disease of suids, known to severely damage commercial pig production and infect free-living wild boar in Europe (1). It is recognized by the World Organization for Animal Health (OIE) as a notifiable disease and its circulation has an impact on international trade. There is, therefore, considerable interest in detailed assessments of the risks posed by this disease to help countries prepare for its incursion, and the required contingency responses. These responses include those affecting commercial pig farms, as well as back-yard producers rearing small herds and some interventions produce an economic burden *per se*; limiting the duration of these controls is of economic interest. Where the disease may circulate in wild hosts, the risks this produces to commercial pig production must be also be considered. The description of these risks includes the character of outbreaks in wild boar (i.e., duration, intensity, geographical extent) where free-living populations overlap with commercial or back-yard pig premises. Predicting what an outbreak of ASF in wild boar might look like can also help inform the design of policies to address these risks. Such policies should be proportionate and practical (2), following available guidelines (3, 4) and should be based on the best available science (5, 6).

The spread of ASF westwards across Europe has been thoroughly described and analyzed to support both local action and to adhere to international agreements on actions and trade (7). There have been several studies using simulation models to explore the relative benefits of a variety of management responses to the detection of ASF in wild boar [e.g., (8, 9)]. All these studies assume a widespread distribution of wild boar across extensive regions of Europe, which are often densely forested and/or sparsely inhabited.

The current guidance produced by EFSA for the management of ASF in wild boar lists actions to contain the infection to a geographically discrete population of wild boar until the infection “burns-out” (defined here to mean a situation with zero infected animals) within this prescribed zone, using intensive focal hunting and a non-hunted buffer to prevent the propagation of disease across the wider landscape (7). In parallel, active searches for wild boar carcasses must be carried out to reduce onward infection. This policy has proven effective in one of the more recent outbreaks in the Czech Republic (Zlín area), where the infection in wild boar has now been declared absent (https://europa.eu/rapid/press-release_MEX-19-1431_en.htm).

The situation in England differs sufficiently from that of continental Europe to suggest that additional predictive modeling is required to inform decision-making. In particular, differences include the character and ownership patterns of the landscapes supporting boar, along with attitudes toward them. The first of these (patterns of landscape) influences the demographic and spatial dynamics of the wildlife host, potentially altering the course of an unmanaged outbreak of ASF from that anticipated in Europe; whilst the latter (attitudes) may affect the acceptability or effectiveness of potential management tools. In England, landscapes commonly host fewer, smaller and more fragmented forested areas than those typically found in Europe, and are dominated by a fine-scaled mosaic of arable land and pasture interspersed with small woodlands, people, and infrastructure (e.g., settlements, major roads, canals), all of which are likely to modify the movement behavior of individual boar as well as their population processes (population dynamics). The recent reintroduction of boar and the landscapes available to them have to date restricted them to a few discrete populations. The most significant of these, in the Forest of Dean, is thought to have only established relatively recently (10) and appears to still be spreading and increasing in density in its core.

To better understand the risks to commercial pig production posed by the incursion of ASF into a free-living population of wild boar in England (high density but spatially isolated) we developed a spatially explicit individual-based model (IBM) and used it to predict the course of disease in wild boar and explore the value of different prophylactic and reactive management actions to mitigate the risk. Specifically, we wished to test the hypothesis that the risks produced by ASF would be similar to those identified for foot-and-mouth disease (FMD) (11), and required a detailed prediction of the duration of disease circulating exclusively within the population of free-living wild boar (and the consequent continuous presence of uncontained or unmanaged ASF virus in the landscape). We then wished to evaluate the significance of population size (boar abundance and distribution) and the value of prophylactic population control in dictating the severity of an outbreak, and compare this to a number of reactive tools (after the discovery of disease; increased levels of culling and carcass retrieval) to most quickly, or effectively achieve disease elimination.

## Methods

### Study Site

**Figure 1:**
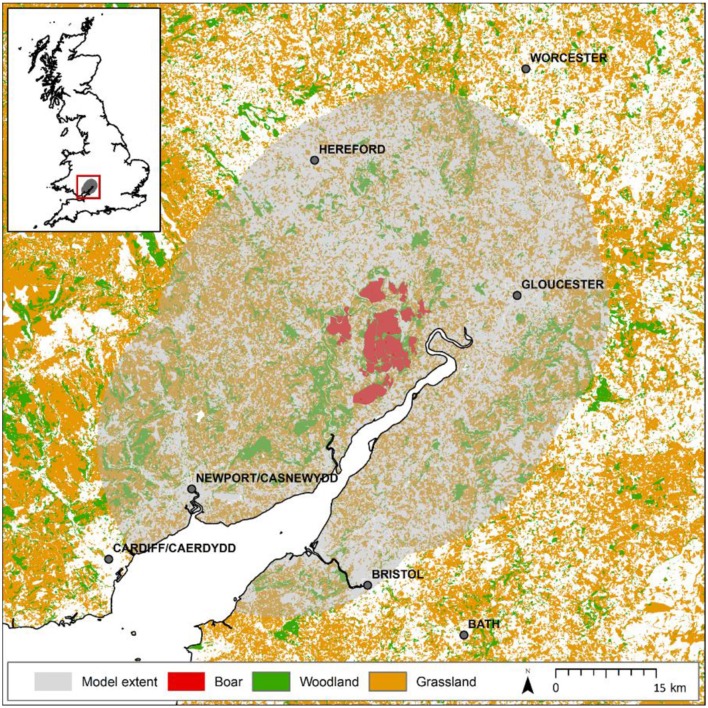
Map of study extent around the Forest of Dean. Model considers a 25 km buffer around the forest estate separated into two regions: that currently monitored (red), where boar are known to be present and are controlled, and that unmonitored (shaded) where anecdotal evidence suggests boar are yet to establish possibly due to hunting activities. Presumed habitats suitable for boar (woodland and grassland) are shown demonstrating the quality of the wider landscape to support species expansion. The map shown in this figure contains data (GB coastline) obtained from the OS Strategi™ dataset 2016. This data is freely available under an open government license (©Crown copyright and database rights 2016). Land cover data (woodland and grassland) is based upon LCM2007© NERC (CEH) 2011 made available to Defra under license. LCM data also contains Ordnance Survey data© 2007. For full details of the LCM dataset [see Mortan et al. (12)].

The Forest of Dean (e.g., 51.80°N, 02.52°W) is located in south-western England. It includes an extensively forested core area owned by the Forestry Commission (hereafter the FC estate) comprising a fragmented 75 km^2^ of mixed woodland surrounded by a mosaic of arable, pastures and smaller woodlands largely in private ownership (Figure 1). Our study extent is defined by a 25 km buffer around the FC estate in the Forest of Dean and describes an arena of ~4,500 km^2^.

Feral wild boar was first detected on the FC estate during the 1990's and has been monitored annually since 2013. Population management using targeted culls (restricted to the FC estate) has been ongoing since 2008 (13). Latest figures published by Forest Research suggest that the current population is 1,635 (14); an increase of 60% from figures reported in 2015 (15), despite removal of ~500 individuals per year (14). Information about the distribution or abundance of feral wild boar in the wider landscape is unavailable, as is any empirical description of the hunting effort or hunting bag size.

### Model Framework

#### Overview

The model was adapted from that outlined in Croft et al. (11), previously developed to explore management of foot-and-mouth disease (FMD) in wild boar. Written in the Python 2 programming language (16) (code files provided in Supplementary Material), this model applied an individual-based approach with agents (individual wild boar) operating across a simulation of a real-world landscape. Agents were distinguished by their demographic class (e.g., age, sex) and exhibited defined stochastic behaviors (e.g., survival, reproduction, dispersal) in order to emulate a realistic population and spatial dynamics. The avoidance of a grid-based model has been shown to reduce bias (17, 18). In addition, the model also included processes to represent management activity applied to remove boar within the FC estate (removal through variable effort—culls to achieve fixed target quotas) and the wider landscape (unregulated hunting without descriptions of effort or effect). In the UK, both culling and hunting rely on shooting free ranging animals and are functionally identical. We distinguish between them here for clarity of inference and discussion by defining culling as the organized and coordinated activity in one area to achieve a policy objective, and hunting as the unmanaged, unregulated and unmonitored *ad-hoc* activity across the wider study arena. To simulate these activities, animals were removed from the model with fixed weekly probability (hereafter referred to as p. culled and p. hunted, respectively). Full details of the core host model are available in Croft et al. (11).

The main modifications relate to the model's epidemiological component to simulate the key characteristics of ASF. Following Lange and Thulke (8), we consider direct transmission through contact with infected conspecifics within the same social group and through contact with infected carcasses distributed across the landscape. To simulate carcass mediated transmission, individuals that die as a result of disease are retained in the environment as a source of infection with which living conspecifics in the deceased individual's current social group (patch) and up to one neighboring social group (determined according to the relative proportions of range overlap) can interact (8), becoming infected according to a fixed probability. Carcasses remain in the environment for a fixed period after which they are no longer considered a source of infection and are removed. The small number of boar which survive ASF are considered to become immune for life; females beginning lactation within 15 weeks of their initial infection can convey maternal immunity to their dependent offspring until the end of this period (Table 1), though these subsequently become susceptible once independent.

**Table 1:** Epidemiological parameter values used in the model.

**Parameter**	**Value**
Probability of infection (conspecific)	0.05
Probability of infection (carcass)	0.15
Group overlap distance (km)	1.35 km
Period from infection to death	1 week
Persistence of maternal antibodies	15 weeks
Disease-induced mortality (individual)	0.95
Disease-induced mortality (pre-natal mortality)	0.5
Disease-induced fertility reduction	0.625

*ASF specific parameter values adopted from Lange and Thulke (8)*.

#### Parameterization

The boar components of the model were parameterized as described in Croft et al. (11), based on values from existing literature, empirical studies and other models. Epidemiological parameters were adopted from Lange and Thulke (8) and are detailed in Table 1. All were applied directly, with the exception of the probability of disease-induced death in the core areas of a patch, i.e., distinct non-overlapping area where conspecifics in neighboring social groups will have no contact with carcasses.

Lange and Thulke (8) define this factor based on an interaction between 3 × 3 km resolution cells as the central 1% or 300 × 300 m of each cell (or patch). Here, we generalize this representation by computing the corresponding width of overlap between neighboring social groups (1.35 km either side of a boundary line) which, when applied to the irregular polygons we use to portray our real-world landscape (mean area 3.9 km^2^), can be used to derive the probability that contact with a carcass will remain exclusively within a social group.

#### Simulations

Populations were initialized according to a fixed distribution of boar approximating that reported for the FC estate in 2015 (15). Initial demographics were applied to match the stable structure achieved following a 5 year burn-in period (running the model prior to the main simulation including disease and management) (11). For each parameterization, we performed 100 unique repetitions [10 repetitions for each of 10 different randomized representations of boar social grouping across the landscape; refer to (11)] from which summary statistics were produced. Specifically these were: change in total population (boar), area occupied (km^2^), density (boar/km^2^) and, for simulations with ASF introduction, the time to disease freedom (weeks) and change in the cumulative size of infection; the latter represented by both the linear distance between the centroids of the patch hosting the focal case and the most distant patch of cumulative infection (km) and the cumulative area infected (km^2^).

### Disease Scenarios

Using our model we simulated different outbreaks of ASF, infecting an individual selected at random, assuming various timings of release (after 0, 3, 5, 10, 15, and 20 years), which produces a range of simulations run across populations of differing size and distribution. In this study, infection always occurred on the same day of the year (representing the 15th April), producing a seasonally fixed response. For each of these scenarios we tested the efficacy of different management options combining both removal (culling and hunting) and environmental decontamination (carcass retrieval). Initially, we varied hunting rate (p. hunted) with a fixed rate of culling (p. culled) within the FC estate equivalent to that applied in 2015 [p. culled = 0.0065; weekly probability of removal (11)] considering: (i) where hunting is completely absent (p. hunted = 0); (ii) where its rate matches the rate within the FC estate (p. hunted = 0.0065); (iii) where hunting is so efficient it immediately removes any boar that appear in the wider landscape (p. hunted = 1). We also considered scenarios exploring intensified culling rates within the FC estate, specifically a 50 and 100% increase in weekly removal probability (p. culled = 0.01 and 0.013, respectively), assuming a fixed rate of hunting (p. hunted = 1).

We then considered the addition of carcass retrieval to control an outbreak by shortening the time that carcasses remain in the model. For each management scenario using removal alone, we tested reducing carcass persistence from 8 weeks [reflecting the average time mainly invertebrate scavengers take to completely consume a carcass (19)] to either 4 weeks or 2 weeks to reflect moderate and intense search and removal efforts.[Based on the time to disease eradication (zero infected individuals), we compared results to assess the efficacy of both prophylactic (long-term population control prior to disease introduction) and reactive control options (increased removals and environmental decontamination). It should be noted that in all simulations disease detection occurs quasi-simultaneously with release. As a consequence of this choice, the efficacy of reactive control options tested should be interpreted as an absolute “best case” intervention when an outbreak is at its weakest.

## Results

**Figure 2:**
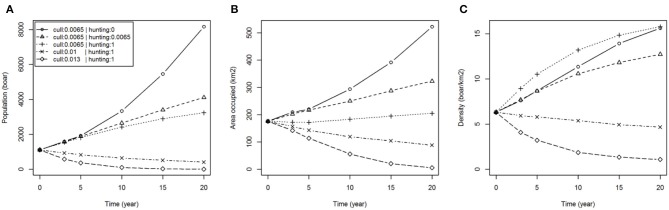
Boar population dynamics under various hunting and culling scenarios. Plots show: **(A)** total population (boar); **(B)** area occupied (km^2^); **(C)** density (boar/km^2^); over time for different hunting scenarios (none: p. hunted = 0, equal to current culling: p. hunted = 0.0065 and immediate removal: p. hunted = 1) with fixed culling (p. culled = 0.0065) and different culling scenarios (50 and 100% increase in culling rate: p. culled = 0.01 and 0.013, respectively) assuming containment (immediate removal from hunting: p. hunted = 1).

The levels of culling and hunting affect the size and distribution of the population of wild boar over time (Figure 2). If no hunting were to occur outside of the FC estate (p. hunted = 0) then within 20 years the wild boar population could reach around 8,000 individuals (more than 7 times the population in 2015) occupying 500 km^2^ (more than double that of 2015). With moderate hunting (p. hunted = 0.0065), equal to the estimated culling rate in the FC estate [i.e., ~45% of the population removed per year (15)], our results showed that wild boar populations would still grow but at a slower rate, reaching nearly 4,000 individuals occupying 300 km^2^ in the same 20 year period. With intense and continuous hunting (immediate removal of animals in the landscape beyond the FC estate i.e., p. hunted = 1) wild boar populations would continue to grow within the FC estate until reaching a self-regulating carrying capacity at a mean density of 15 boar/km^2^ (~3,000 boar occupying the 200 km^2^ of the FC estate and immediately adjacent land) but would not establish in the wider landscape. Extending the latter scenario to consider increased culling rate within the FC estate successfully reduced population size and distribution, albeit with declines occurring slowly over the 20 year course of the simulations (Figure 2). Eradication of wild boar within 20 years might be achieved if populations were contained and the culling quota within the FC estate were doubled, although we cannot specify the effort that would be required to sustain such a high rate of removal at low densities of boar.

**Figure 3:**
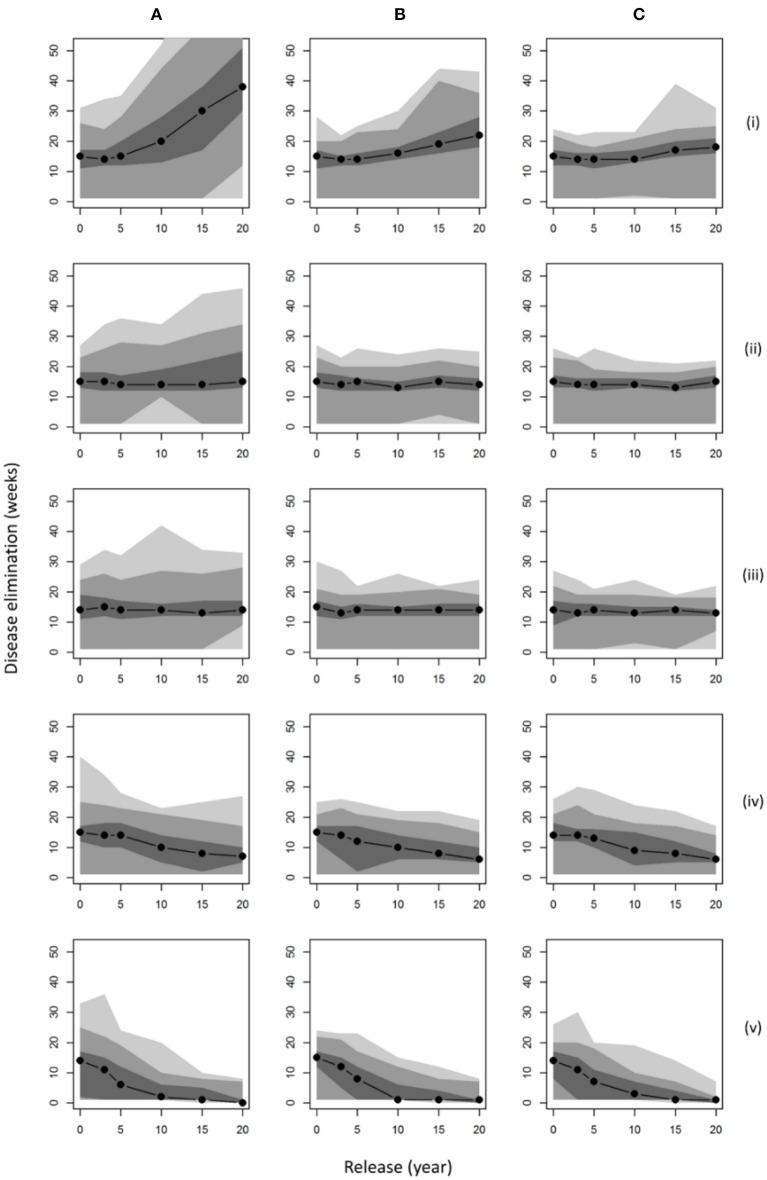
Disease persistence under various management scenarios. Plots show the median time to disease elimination (zero infected individuals) given different points of initial release assuming: **(A)** no carcass retrieval (8 week persistence); infected carcasses retrieved within **(B)** 4 weeks; **(C)** 2 weeks; and population control applying: current culling (p. culled = 0.0065) on the FC Estate with (i) no hunting (p. hunted = 0); (ii) identical hunting (p. hunted = 0.0065); (iii) immediate removal from hunting (p. hunted = 1); on surrounding land; (iv) 50% additional culling (p. culled = 0.01); (v) 100% additional culling (p. culled = 0.013); both with immediate removal from hunting (p. hunted = 1). Shaded regions denote smoothed ranges centered on the median containing (from darkest to lightest): 50, 90, and 100% of model repetitions.

Considering the time to disease elimination for each of these management scenarios (Figure 3), we observed a notable positive relationship between population size (abundance and distribution; Figure 2) and the persistence of disease in the landscape. For a wild boar population similar to that estimated in 2015 (~1,500 individuals), our results predicted a median time to elimination of disease of around 15 weeks. If populations were allowed to grow uncontrolled across the wider landscape (hunting = 0) for 20 years, an outbreak of ASF could last nearly 3 times longer (median 40 weeks) with the un-managed disappearance of disease (no reactive controls such as carcass retrieval) becoming very unlikely to ever occur within 10 weeks (<5% probability). It is only in populations of wild boar hunted into decline that burn-out is predicted to occur in <10 weeks, i.e., scenarios (a) (iv) and (a) (v) (Figure 3) where culling was increased within the FC estate whilst preventing dispersal to the wider landscape (p. hunted = 1). However, such measures would not prevent the outbreak of disease unless boar populations were very small (<100 boar) at the point of introduction. Comparing the impact of carcass retrieval (Figures 3B,C), our results showed only marginal shortening of the time to disease elimination, except in the case when initial populations are largest where this environmental decontamination could nearly halve outbreak length when retrieval is within 4 weeks (from 40 to 20 weeks). Increasing effort further to ensure retrieval within 2 weeks showed no additional benefit. Whilst carcass retrieval did not impact median times to disease elimination it did limit the likelihood that outbreaks would last substantially longer than the median duration.

**Figure 4:**
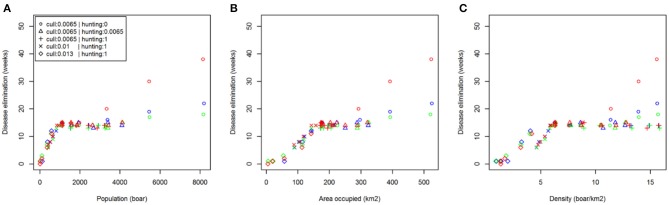
Relationship between disease persistence and population structure. Plots show median time to disease elimination (zero infected individuals) against: **(A)** total population (boar); **(B)** area occupied (km^2^); **(C)** density (boar/km^2^); at the time of initial disease introduction. The color of symbols denotes different carcass retrieval strategies: (red) none; (blue) 4 weeks; (green) 2 weeks.

Comparing scenarios with identical initial conditions, we assessed the effect of reactive population control rather than prophylactic reductions prior to disease introduction (Table 2). These results showed that increasing the rate of management (culling or hunting) in response to ASF has no effect. As already stated, carcass retrieval does not reduce median outbreak length but does reduce the likelihood of more extreme events (i.e., exceptionally long outbreaks). This result was supported by examining the relationship between time to ASF elimination and initial population size, distribution and density across all management options (Figure 4).

**Table 2:** Results of potential responsive control options.

**Culling (p. culled)**	**Hunting (p. hunted)**	**Elimination (weeks)**	**Scenario (Figure 3)**
**Responsive hunting**			
0.0065	0	15 (1, 20)	(A) (i)
0.0065	0.0065	15 (1, 21)	(A) (ii)
0.0065	1	14 (1, 22)	(A) (iii)
**Responsive culling**			
0.0065	1	14 (1, 22)	(A) (iii)
0.01	1	15 (1, 23)	(A) (iv)
0.013	1	14 (1, 23)	(A) (v)
**Carcass retrieval (2 weeks)**			
0.0065	0	15 (1, 24)	(C) (i)
0.0065	0.0065	15 (1, 21)	(C) (ii)
0.0065	1	14 (1, 24)	(C) (iii)
0.01	1	14 (1, 24)	(C) (iv)
0.013	1	14 (1, 25)	(C) (v)

*Median time to disease elimination (zero infected individuals) for various responsive management options simulating the release of disease in 2015 (year 0). Brackets denote 5 and 95% CI*.

**Figure 5:**
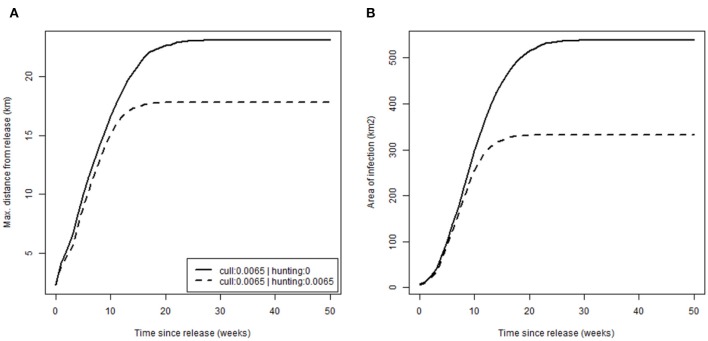
Rate of disease spread. Plots show cumulative disease spread based on: **(A)** the maximum distance from release (km); **(B)** the area of infection (km^2^); over time for different hunting scenarios (none: p.hunted = 0 and equal to current culling: p.hunted = 0.065).

Finally, we evaluated how management, specifically hunting, alters disease spread by comparing rates of spread (Figure 5) in the absence of hunting [scenario (a) (i), p. culled = 0.0065 and p. hunted = 0, with release after 20 years; Table 2] against, and with, moderate hunting pressure [scenario (a) (ii) where p. hunted = 0.0065; Table 2]. Patterns of spread were similar with expansion at an initial rate of ~1 km/week by distance, 20 km^2^/week by area, before rapid reduction to zero as infection reached the limit of the boar distribution. Hunting did appear to slow disease spread but only marginally, perhaps as a result of a lower boar density (13 compared to 15 boar/km^2^ when no hunting was applied). Similarly, toward the edge of boar distributions, where densities were closer to that observed in Europe (5 boar/km^2^), the rate of spread reduced to ~0.5 km/week, half of that of the core, where densities were substantially higher.

## Discussion

In this study we have outlined a spatially-explicit individual-based model of wild boar, incorporating a novel description of the underlying model landscape used to simulate our real-world example. We applied this model to predict the epidemiological consequences of introducing ASF to a wild boar population and extended this to explore a wide variety of prophylactic and reactive management strategies. Here we suggest that ASF is unlikely to persist and circulate within this wildlife host indefinitely (i.e., become a self-sustaining endemic disease in wildlife), though we note that our prediction is specific to a discrete and limited population of wild boar in the Forest of Dean. This stands in contrast with the prediction made for FMD in the same landscape (11). In all of our simulations here, even those where boar is projected to have spread for 20 years, ASF is likely to spontaneously disappear, limiting the duration of the risk this produces to commercial pig production. However, in the scenarios in which the population of boar is most extensive, disease might continue to circulate for up to 40 weeks, which compares to burn-out in a median of 15 weeks for more contemporaneous simulations of disease.

Unlike previous modeling studies [e.g., (8, 26)], which have focused on mainland Europe, where wild boar are ubiquitous (25), we consider an isolated and expanding population typical of that found in England, and estimated to contain ~1,500 individuals at densities up to 20 boar/km^2^ (10). However, without near eradication of the host population (geographically restricted populations with densities below 2 boar/km^2^) few of the tested reactive management responses shorten the length of an outbreak to <14 weeks. Reactive controls, including increased culling and carcass retrieval, have negligible impact in this context, only showing notable improvements in the most extreme scenarios (halving the time to elimination in the largest populations from 40 weeks to 20 weeks). Similar to previous work (11) on foot-and-mouth disease (FMD), our results suggest that an important factor predicting the severity (duration) of an outbreak, and therefore the key to reducing it, is not population density as may be expected, but the extent of the wildlife host distribution (occupancy across the landscape). However, we also note a potential relationship between density and the rate of disease spread, which if reduced sufficiently may inhibit the progress of outbreaks in some instances.

### Model Validation

Other than our description of ASF, which for this study follows the relatively deterministic approach of studies supporting the current EFSA guidance (26), there are considerable uncertainties in simulating the demography and behavior of wild boar in a real-world context. The limited quantity and quality of available data or similar examples of focal outbreaks in isolated wild boar populations makes this approach very difficult to validate. Evidence of the utility of our model, therefore, rests on the quality of its component elements (i.e., representation of the landscape, the host wildlife population process, the simulation of host wildlife movement, epizootiological rates) and their coherent integration into the model system. We achieved this by selecting approaches known to minimize the introduction of bias and which simulate, as directly as possible, the biology and behavior of the wildlife host in real landscapes. Unlike other models (8, 26), which use a grid-based structure, our model landscape (a continuous mosaic of patches of irregular geometry scaled precisely to socially coherent sub-populations wild boar; sounders) allows us to represent real-world locations without bias (17), capturing the fine scaled variation in land-use (composition and configuration of habitats) and the heterogeneity of their value to boar, their movement (accounting for any natural barriers such as major roads, rivers, and canals), and as a consequence, the spatial heterogeneity in contact rates. Likewise, our choice to use an IBM approach allows us to capture individual variation in movement (e.g., dispersal of juveniles, stasis of boar in favorable locations), as well as permitting us to consider population processes in small populations without bias. For example, in our simulations, sub-populations in each patch are often small or very small, either because their local environment (patch) may not support more boar, or because they are at the spreading edge of an expanding population. Similarly, ASF is so infectious that variations in outbreak outcomes can be dictated by the fate of individual boar; such as stochastic mortality inhibiting disease spread, or relatively rare long-distance movement by individuals promoting disease spread.

Despite the lack of data on ASF in English wild boar, we are able to compare an emergent property of our epizootiological model with descriptions produced in other studies. The model identified a rate of spread of ASF of ~4 km/month within the core population at densities around 15 boar/km^2^, reducing to nearer 2 km/month toward the population edge where densities were closer to that observed in Europe (5 boar/km^2^). This compares well with an empirical description of 1.5 ± 1.3 km/month for the same disease in boar in Poland (27), 1–2 km/month in a number of eastern European countries (24), and a broader range of estimates for the unassisted spread of ASF cited in (7) between 8 and 25 km/year, though most fall between 10 and 17 km/year (0.8–1.4 km/month). Whilst not a definitive validation of our model, the similarity of our result with that of others suggests that our representation and parameterization of our model system is of some value and is free of substantial consistent bias.

### Disease Predictions

In all of our scenarios, even those simulating relatively large and extensive populations of boar [e.g., scenario (a) (i) after 20 years; Table 2], ASF fails to produce a self-sustaining disease. Infection spreads rapidly wherever boar occur in our simulated landscape, causes substantial and rapid mortality, and is predicted to eventually disappear (“burn-out”). Given our choice to model a virulent strain of the virus (95% mortality) and a geographically isolated population, the duration of ASF in the landscape represents the time it takes disease to physically spread to every occupied patch. This was illustrated by the close correlation of outbreak duration to a measure of distribution (Figure 4B). We anticipate that once the virus arrives at a patch, the virulence of the disease and the social behavior of the boar will reduce its sub-population in a relatively short time. Model assumptions and the rapid progress of disease make it unlikely that recruitment has introduced a substantial cohort of new susceptible boar into our system during an outbreak (excepting a few scenarios), and immigration can also be discounted in this study. These latter processes must partly explain the apparent endemicity of ASF at large scales in extensively forested European landscapes. Some scenarios in this study [i.e., scenario (a) (iii) where p. culled = 0.0065 and p. hunted = 1] replicate the consequence of the current EFSA recommendation to create and maintain a buffer around an infected core zone in landscapes where boar are widespread (1). Our model suggested that if activities that might induce long movements by potentially infective wild boar are avoided, ASF should eventually disappear from small core zones (e.g., <500 km^2^).

Both the demographic and spatial dynamics of boar vary seasonally, mainly in relation to food availability, in ways that might alter the course of disease outbreaks. For example, plentiful food in autumn might reduce or delay density-dependent dispersal between patches (temporarily dampening the source-sink spatial dynamic) (21) or temporarily reduce ranging within patches, lowering inter-patch contact rates. Conversely, the presence of taller crops in arable fields might temporarily promote movement within- and between- patches (enhance the spatial dynamic). Outbreaks of disease, which persist throughout seasonal variations in movement may produce distinct epizootiological dynamics. The fixed annual date of the disease scenarios applied here may have failed to catch some of this variation, as the disease in our simulations will have systematically removed most of the birth pulse, and burnt-out before most juveniles were considering dispersal (22). Future work could explore varying the date of the focal infection, to explore the effect of recruiting susceptible individuals during an outbreak (likely to lengthen outbreaks), seasonal variation in the spatial dynamics of boar, seasonal variation in the removal of boar, or a seasonal variation in the virulence of the disease in boar. However, given the short duration of the epidemic in most circumstances, we do not believe this is likely to change the probability of ASF becoming endemic.

### Implications for Risk Assessment and Contingency Planning

In agreement with Croft et al. (11), this study suggests that the distribution of boar is a useful predictor of the duration of an outbreak of a highly contagious disease in a closed population. The utility of this finding recognizes that establishing the distribution for a large ungulate prone to leaving obvious activity signs is relatively inexpensive and quick compared to the effort required to measure abundance or density. We suggest that risk assessments of the impact of ASF might be possible using the distribution of a population, potentially informed by citizen scientists; although the quality of that description and the risk assessment it underpins would be substantially improved by extending the data to include quality assured observations and some element of geographically targeted and/or systematic descriptions of distribution (23).

We explored the importance of differing cull and hunting rates and the effect these have on the duration of an ASF outbreak. Our implementation of this (a per capita risk of removal) simulates the removal of boar in direct proportion to their density homogenously across the landscape. The culling and hunting rates do not describe the effort needed to remove wild boar. Thus, we do not consider the additional effort necessary where densities of boar are low or the terrain difficult (e.g., dense forests or areas close to people). Whilst the additional costs and complexities of delivering an effective annual cull across the difficult terrain of the FC estate is borne by its state owner, there is likely to be substantial spatial variation in the rate of hunting by private landowners. This heterogeneity is likely to perturb natural source-sink dynamics between patches, either directly through disturbance or indirectly by stimulating movement produced by density-dependent dispersal, and consequentially leading to increased spread of disease. This principle has been recognized in the advice given to EFSA on the control of ASF in areas of Europe where wild boar are widespread, for member states to ban hunting close to the focus of disease (1). Unlike some previous studies [e.g., (9), we did not associate high rates of removal with an increased rate of dispersal despite some evidence that this can occur (20, 28, 29), as our choice recognizes that removal in England will almost certainly be restricted to shooting. We also make an additional important distinction between the functionally similar culling and hunting. Culling, as we use it here, encapsulates not only the coordinated and targeted action to achieve a pre-defined target, but also the professionalism of contracted specialists who can be required to fulfill complex directions in official guidance (1) and remove animals with minimal disturbance. This contrasts with our use here of the term hunting, to describe an un-managed activity where variations in the interests of landowners may drive variations in hunter behavior (e.g., for sport, to protect property, for commercial gain), some of which may be problematic. For example, private hunters shooting free ranging boar commonly use sites baited with supplementary feed; an activity which might promote boar population growth because bait points are overstocked (30), produce conflicts with other biodiversity (31), or may act as points of enhanced disease contact (30). Importantly the heterogeneity in hunting rate will also be conditional, with some landowners objecting in principle to any hunting, effectively providing refuges for boar; across our study arena we estimate there to be at least 6,200 separate landowners (mean ownership of 0.55 km^2^ ranging up to 130 km^2^). In reality, it is unlikely that the perfect hunting we simulate here (hunting = 1) could ever be realized in landscapes with complex fine-scale patterns of private ownership and variable densities of boar, and that target rates for hunting would need to be set appropriately high to realize any policy to prophylactically control wild boar. This highlights a critical gap in our knowledge of the costs (efficiency per hunting day) and consequences (disturbance) of hunting boar in England, how this is affected by the variation in method of hunting, mode (intensity and frequency), or the varying interests of landowners.

The model suggested that in all scenarios median disease burn-out occurred around week 14–15. The only benefits of an ongoing contingency response (the removal of boar beyond the FC estate, doubled culling rates within the FC estate and carcass removal within 2 weeks) are a reduction in the likelihood of long outbreaks (worst case) lasting more than 20 weeks. However, in this context, we note that our estimates of culling rates or their integer multiples, whilst “realistic” and derived from FC estate cull operations (10, 11), are still small when applied on a weekly basis and are likely to produce little effect as a response within the short duration of most disease outbreaks.

Similarly to other authors (26, 32), we show that the removal of carcasses as a contingency response to the outbreak of disease has little value in shortening the median duration of the infection in wild boar for many of our scenarios. Only where boar have become relatively widespread in our simulations [scenario (a) (i) vs. (a) (iii); Table 2] does the considerable effort of retrieving carcasses within 2 weeks appear to be of substantial value in changing the course of an outbreak. However, removing carcasses may have other benefits and might still be considered as a prudent tool in response to disease. For example, the considerable persistence of virus in decaying boar produces a number of risks resolved by the collection of carcasses, such as the accidental movement of contaminated fomites into commercial pig units or backyard pigsties by man or wildlife. Our choice not to simulate the short-term intensive culling, such as that applied within the Czech outbreak (https://europa.eu/rapid/press-release_MEX-19-1431_en.htm), was motivated by the observation that disease “burn-out” is relatively rapid and is likely to overtake the sum of delays produced by the detection and confirmation of disease, the deployment of assets to manage the disease in wild boar (financial, staff and equipment, legal permissions), and the efficient operation of an intense responsive cull.

## Concluding Remarks

This study predicts that ASF introduced to the free-living wild boar is very unlikely to become endemic in the Forest of Dean for some time, in contrast to the predictions made for FMD. Consequently, the duration of the risks to commercial pig production resulting from uncontained and unmanaged ASF virus circulating in this English landscape is limited. The last new infection of wild boar is likely to be around 15 weeks after the focal infection, and active disease in wild boar rarely persisted for more than 25 weeks in any individual simulation. This outcome is likely to be consequent on the spatially discrete (isolated) and limited size of the population of wild boar, even in scenarios projected after 20 years of population growth and natural spread. This and earlier work (11) both suggest that the persistence of an exotic disease in an isolated population is dependent on the total distribution of the population, rather than the population size *per se*. We suggest that measures of the distribution of boar (e.g., occupancy) are the easiest measures by which the duration of disease circulating in wild hosts can be quickly assessed. Here, all of the reactive management responses appear to have limited value in reducing the duration of ASF in wild boar, though in part that is a consequence of the rapid burn-out of the disease in free-living boar even without intervention. We also explore the potential value of prophylactic management of boar populations, and outline some of the issues which may need to be considered if this is to be adopted as a disease management strategy. These include the distinctions between organized culls undertaken as a professional activity and voluntary hunting in its varied forms. The potential for mismatches between the local density of boar and the spatial heterogeneity in management effort, as well as how management itself may affect the behavior of boar are identified as substantial gaps in knowledge.

## Data Availability Statement

All datasets generated for this study are included in the article/Supplementary Material.

## Author Contributions

GS, JA, and SC conceived the main research idea. SC designed the methodology with contributions from JA. All authors contributed critically to the analysis and interpretation of the results, to the writing of the manuscript, and contributed to sourcing and collating of key input data.

### Conflict of Interest

The authors declare that the research was conducted in the absence of any commercial or financial relationships that could be construed as a potential conflict of interest.
